# Association between Parkinson’s Disease and Diabetes Mellitus: From Epidemiology, Pathophysiology and Prevention to Treatment

**DOI:** 10.14336/AD.2022.0325

**Published:** 2022-12-01

**Authors:** Haiyang Yu, Tong Sun, Xin He, Zhen Wang, Kaidong Zhao, Jing An, Lulu Wen, Jia-Yi Li, Wen Li, Juan Feng

**Affiliations:** ^1^Department of Neurology, Shengjing Hospital of China Medical University, Shenyang, Liaoning, China.; ^2^Department of Pediatrics, Shengjing Hospital of China Medical University, Shenyang, Liaoning, China.; ^3^Laboratory of Research in Parkinson’s Disease and Related Disorders, Health Sciences Institute, China Medical University, Shenyang, Liaoning, China.; ^4^Neural Plasticity and Repair Unit, Department of Experimental Medical Science, Lund University, Lund, Sweden.

**Keywords:** diabetes mellitus, Parkinson’s disease;, epidemiology, pathophysiology, antidiabetic drugs, treatment

## Abstract

Diabetes mellitus (DM) and Parkinson’s disease (PD) are both age-related diseases of global concern being among the most common chronic metabolic and neurodegenerative diseases, respectively. While both diseases can be genetically inherited, environmental factors play a vital role in their pathogenesis. Moreover, DM and PD have common underlying molecular mechanisms, such as misfolded protein aggregation, mitochondrial dysfunction, oxidative stress, chronic inflammation, and microbial dysbiosis. Recently, epidemiological and experimental studies have reported that DM affects the incidence and progression of PD. Moreover, certain antidiabetic drugs have been proven to decrease the risk of PD and delay its progression. In this review, we elucidate the epidemiological and pathophysiological association between DM and PD and summarize the antidiabetic drugs used in animal models and clinical trials of PD, which may provide reference for the clinical translation of antidiabetic drugs in PD treatment.

## 1.Introduction

Diabetes mellitus (DM), the most common chronic metabolic disease globally, has become a heavy public health burden because of its high prevalence and associated disability and mortality [1]. DM is a heterogeneous group of disorders characterized by hyperglycemia and glucose intolerance. It mainly comprises two subclasses: type 1 diabetes mellitus (T1DM) (insulin-dependent diabetes) and type 2 diabetes mellitus (T2DM) (noninsulin-dependent diabetes). Its progression is accompanied by several complications including vision loss, stroke, kidney failure, heart attack, leg amputation, and nerve damage [2]. Parkinson’s disease (PD) is the second most prevalent neurodegenerative disease following Alzheimer’s disease (AD). PD is characterized by typical motor symptoms, including resting tremor, bradykinesia, rigidity, and postural disability. Other nonmotor symptoms such as constipation, anxiety, depression, and rapid eye movement sleep behavior disorder (RBD), can also be present before or during the occurrence of motor symptoms [3, 4]. Recently, the association between DM and AD has been intensively studied. Researchers have recently proposed AD as ‘type 3 diabetes mellitus (T3DM)’ because of the overlap of risk factors and pathophysiological mechanisms [5]. Considerable evidence has confirmed the association of T2DM with memory decline and cognitive impairment. Insulin resistance is the core characteristic of T2DM that contributes to AD and related dementias (ADRDs) [6]. However, the association between DM and PD remains to be elucidated.

## 2.Epidemiological association between DM and PD

Both DM, particularly adult-onset DM (T2DM), and PD affect the aging population, with rising prevalence due to increasing longevity. In the past three decades, the prevalence of DM has increased substantially with approximately 422 million people affected worldwide. A recent study showed the prevalence of DM in China and the US to be 11.2% and 14.3%, respectively [7, 8]. PD prevalence is generally estimated at 0.3% of the entire population and approximately 1% among people over 60 years old, increasing the burden on patients’ families and society [9-11].

**Table 1:** Association between DM and PD in some epidemiological studies and meta-analyses.

Authors, pulication year and references	Study types	Main results
Cerada et al., 2011 [12]	meta-analysis	DM is a risk factor for future PD (RR 1.34; 95% CI 1.14-1.58) in cohort studies (n=4).
		in case control studies (n=5), lack of a relationship between PD and DM (OR 0.56; 95% CI 0.28-1.15)
Cerada et al., 2013 [13]	meta-analysis	DM is a risk factor for future PD (RR 1.26; 95% CI 1.00-1.58) in cohort studies (n=5)
Lu et al., 2014 [14]	meta-analysis	in case control studies (n=14), DM have a negative association with future PD (OR 0.75; 95% CI 0.58-0.98)
Yue et al., 2016 [15]	meta-analysis	DM is a risk factor for future PD (RR 1.38; 95% CI 1.18-1.62) in cohort studies (n=7)
Chohan et al., 2021 [17]	meta-analysis	T2DM was associated with an increased risk of PD (OR 1.21; 95% CI 1.07-1.36); T2DM was associated with faster progression of motor symptoms (SMD 0.55; 95% CI 0.39-0.72) and cognitive decline (SMD -0.92; 95% CI -1.50 to -0.34)
	mendelian randomization	a causal effect of T2DM on PD risk (IVW OR 1.08; 95% CI 1.02-1.14); an effect of T2DM on motor progression (IVW OR 1.10; 95% CI 1.01-1.20) but not on cognitive progression
Komici et al., 2021 [16]	meta-analysis	the prevalence of DM in PD patients was 10.02%, (95% CI 7.88-12.16) in observational studies (n=21); DM patients showed a higher risk of developing PD (OR 1.34; 95% CI 1.26-1.43) in observational studies (n=12)
Rhee et al., 2020 [19]	cohort study	Compared with the non-DM group, the adjusted HR of PD was 1.038 (95% CI 1.009-1.067) in the IFG group, 1.185 (95% CI 1.143-1.229) in the DM duration <5 years group, and 1.618 (95% CI 1.566-1.672) in the DM duration >5 years group
S´anchez-G´omez et al., 2021 [18]	cohort study	T2DM and prediabetes were associated with higher risk of PD (HR 1.19; 95% CI 1.13-1.25, and HR 1.07; 95% CI 1.00-1.14, respectively)

PD: Parkinson’s Disease; DM: diabetes mellitus; T2DM: type 2 diabetes mellitus; OR: odds ratio; RR: risk ratio; HR: hazard ratio; CI: confidence interval; SMD: standard mean derivation; IFG: impaired fasting glucose; IVW: inverse-variance weighted method

Recently, some epidemiological studies have shown an association between DM and PD. Many cohort and case-control studies have investigated the association between DM and the risk of PD, and several meta-analyses have been performed. However, the results remain conflicting (Table 1) [12-16]. Prospective cohort studies have suggested pre-existing DM as a risk factor for the onset of PD [12, 13, 15]. However, retrospective case-control studies indicated that DM could decrease the incidence of PD, which is contrary to the findings of cohort studies [12, 14]. This may be due to heterogeneity among studies, confounding factors, and bias such as inclusion and recall biases [12]. In the most recent meta-analysis, Chohan *et al* investigated the association between DM (particularly T2DM) and PD risk and progression. They found that any type of DM slightly increased the risk of PD, but T2DM was associated with an increased risk of PD (odds ratio (OR) 1.21; 95% confidence interval (CI) 1.07-1.36). Moreover, they showed that T2DM was associated with faster progression of motor symptoms, with higher Unified Parkinson's Disease Rating Scale (UPDRS) Part III score (standard mean difference (SMD) 0.55; 95% CI 0.39-0.72), and faster cognitive decline, with decreased Montreal cognitive assessment score (SMD -0.92; 95% CI -1.50 to -0.34) [17]. Furthermore, Sánchez-Gómez *et al* first reported that prediabetes could increase the risk of subsequent PD (hazard ratio (HR) 1.07, 95% CI 1.00-1.14) [18]. Meanwhile, Rhee *et al* found that the risk of PD increased with the increasing duration of diabetes. Compared with the non-DM group, the adjusted HR of PD was 1.185 (95% CI 1.143-1.229) in the DM duration <5 years group, and 1.618 (95% CI 1.566-1.672) in the DM duration >5 years group (Table 1) [19].

In addition to meta-analysis of observational studies, Mendelian randomization (MR) have been used to investigate the causal effect of T2DM on the risk and progression of PD using data from genome-wide association studies (GWAS) [20-22]. MR results suggest a causal effect of T2DM on PD risk and faster progression of motor dysfunction but not on cognitive decline [17]. In conclusion, DM, particularly T2DM as well as prediabetes, play an important role in the risk and progression of PD.

## 3.Pathophysiological crosstalk between DM and PD

Both genetic variants and environmental factors play roles in the pathophysiology of DM and PD. Some DM animal models showed similar pathological characteristics to PD animal models [23]. There are potential mechanisms common to the pathophysiology of both diseases, including misfolded protein aggregation, cell death, mitochondrial dysfunction, oxidative stress, chronic inflammation and microbial dysbiosis [24].

### 3.1Misfolded protein aggregation and cell death in DM and PD

**Figure 1.**
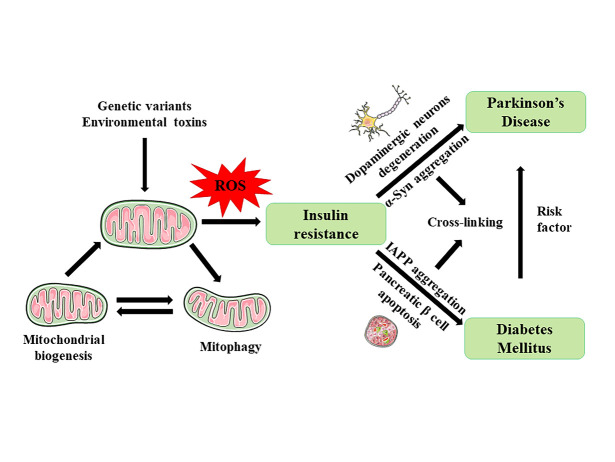
Pathophysiological crosstalk between DM and PD. Various genetic variants and environmental toxins can damage the mitochondria, causing excess ROS production and thereby resulting in insulin resistance. Subsequently, peripheral insulin resistance can cause islet amyloid polypeptide aggregation and pancreatic β-cell apoptosis, and central insulin resistance can cause α-Syn aggregation and dopaminergic degeneration.

Misfolded amyloid protein aggregation and specific type of cell death are involved in both DM and PD. In T2DM, islet amyloid polypeptide (IAPP) or amylin is produced by islet β cells to regulate insulin secretion. Fibrillar IAPP aggregates are the main constituents of protein deposits in pancreatic islets, which cause dysfunction in islet β cells and their death in most patients with T2DM [25]. In PD, misfolded α-synuclein (α-Syn) is aggregated primarily in the cytoplasm of dopaminergic neurons to form Lewy bodies (LBs) or Lewy neurites (LNs), which can cause progressive death of neurons [26] (Fig. 1).

Crosstalk between IAPP and α-Syn has been described previously [27]. Using in vitro cross-seeding experiments, Horvath *et al* found that IAPP amyloids promote α-Syn aggregation and that mixing IAPP and α-Syn monomers results in faster co-aggregation than either alone [28]. In cynomolgus monkeys with T2DM, Sun *et al* found increased aggregation and phosphorylation of α-Syn and partial co-localization with IAPP in the pancreatic islets and brain without obvious dopaminergic neuron death in the substantia nigra (SN), which suggests that T2DM may develop prodromal alterations in PD [29]. In streptozotocin (STZ)-induced T1DM mice models, the proportion of insulin-positive areas to the islet areas in the pancreases of α-Syn-overexpressing mice was significantly lower than that of the wild-type mice, indicating a more severe pancreatic β cell loss in the α-Syn-overexpressing mice [30]. Furthermore, Martinez-Valbuena *et al* found phosphorylated α-Syn deposited in pancreatic β cells in 93% of patients with PD and 68% of patients with T2DM who had no clear neuropathological alterations, and IAPP/α-Syn interactions occurred in patients with pancreatic inclusions of phosphorylated α-Syn [31]. Therefore, co-aggregation of IAPP and α-Syn may be the underlying pathological mechanisms of increased incidence of PD in patients with DM.

### 3.2Insulin resistance and synaptic plasticity in DM and PD

Insulin is a vital hormone secreted by pancreatic β cells to maintain peripheral glucose homeostasis. Insulin functions via insulin receptor (IR), which activates insulin receptor substrates (IRS). Insulin binds to IR/IRS to stimulate various downstream signaling pathways [32]. In addition to its functions in the periphery, it also plays a role in the central nervous system (CNS) [33, 34]. IRs are highly expressed in several brain regions, including the olfactory bulb, hypothalamus, hippocampus, and midbrain, in rats [35, 36]. Insulin resistance is a pathological condition in which cells fail to sense and respond normally to insulin, with decreased insulin/insulin-like growth factor 1 (IGF-1) signaling (IIS). There may be an association between peripheral insulin resistance in T2DM and central insulin resistance in PD [37] (Fig. 1).

Decreased IIS may play a role in the pathophysiology of PD [38]. A cross-sectional study suggested that insulin resistance is higher in the brains of patients with PD [39]. Additionally, IR mRNA levels decreased in the SN from the brains of patients with PD [40]. In the SNpc and putamen of postmortem brain tissue of patients with PD, Bassil *et al* found higher IRS-1-pS312 levels in nigral dopaminergic neurons and a trend toward higher IRS-1-pS312 levels in putaminal neurons but no differences in IRS-1-pS312 staining intensity in glial cells. Moreover, IRS-1-pS312 showed high co-localization with α-Syn within the core of nigral LBs and putamen of PD patients [38]. In PD rat models, IRS-2 phosphorylation at serine 731 increased in the 6-hydroxydopamine (6-OHDA)-induced dopamine-depleted striatum [41]. In α-Syn-overexpressing cells and a mouse model, Gao *et al* found increased IRS-1 phosphorylation at serine 636 and decreased tyrosine phosphorylation, which accelerated IRS-1 turnover and reduced insulin signaling [42]. These results suggest that insulin resistance indeed occurs in PD. In addition, faster disease progression in PD patients with T2DM may be attributed to brain insulin resistance [43]. In ob/ob and db/db T2DM mice, insulin signaling impairment and α-Syn aggregation occurred in both pancreas and midbrain, and T2DM mice were more susceptible to neurotoxicity induced by acute 1-methyl-4-phenyl-1,2,3,6-tetrahydropyridine (MPTP) admini-stration [44]. Similarly, high-fat-diet (HFD)-induced insulin-resistant mice may be more vulnerable to 6-OHDA, which exacerbates nigrostriatal dopamine depletion, vascular alterations, and motor symptoms [45-47].

Moreover, insulin plays a role in regulating brain dopamine concentrations. Insulin can increase dopamine transporter (DAT) mRNA expression in the SN [48, 49]. Moreover, insulin enhances dopamine uptake by activating the phosphatidylinositol-3-kinase (PI3K) pathway and increases dopamine release by activating cholinergic interneurons [50]. Abnormal synaptic plasticity is associated with the motor symptoms of PD. Simultaneously, insulin can regulate synaptic plasticity by controlling synapse density and affecting the formation of synaptic junction and dopamine-mediated synaptic and behavioral plasticity via mammalian target of rapamycin C1 (mTORC1) [51]. These studies provide evidence for brain insulin resistance in PD and support the rationale for repurposing antidiabetic drugs for PD treatment.

### 3.3 Mitochondrial dysfunction and oxidative stress in DM and PD

Different genetic factors and environmental toxins can impair mitochondrial function and subsequently induce oxidative stress, defined as an imbalance between reactive oxygen species (ROS) generation and antioxidant cellular defenses [52]. Although most PD cases are of sporadic type, familial PD accounts for 5%-10% of all patients worldwide [53]. Many familial PD-related genes are involved in mitochondrial function [54]. Autosomal dominant forms of PD are associated with mutations in the SNCA (PARK1/4) gene, encoding α-Syn, and LRRK2 (PARK8), encoding leucine-rich repeat kinase 2. Furthermore, mutations in the Parkin (PARK2) gene, encoding E3 ubiquitin protein ligase parkin, PINK1 (PARK6), encoding PTEN-induced putative kinase 1, and DJ-1 (PARK7), encoding the deglycase protein DJ-1, are involved in the autosomal recessive forms of PD [55]. All these, PD-related genes are related to mitochondrial function. α-Syn can interact with the mitochondrial membrane, causing direct mitochondrial toxicity [56]. LRRK2 mutation inhibits mitochondrial fission and increases ROS production [57]. PINK1 and Parkin participate in mitophagy, and mutations in these genes result in the accumulation of defective mitochondria [58, 59]. DJ-1 is primarily located on the mitochondrial inner membrane and matrix and is translocated to the mitochondrial outer membrane in response to oxidative stress [60]. Inhibition of DJ-1 influences dopaminergic neuron activity in the SN [61]. In addition to genetic mutations, some environmental toxins can cause mitochondrial damage and thereby lead to parkinsonism. Dopaminergic toxins including 6-OHDA, MPTP, rotenone, and paraquat mainly impair the mitochondrial respiratory chain, triggering ROS-induced dopaminergic neuronal apoptosis [62].

In addition, mitochondrial dysfunction plays a central role in insulin resistance [63]. Mitochondria are the primary site of ROS and are critical to energy production, metabolism, redox homeostasis, and multiple cell biological processes [64]. ROS production can activate the phosphorylation of IRS proteins and impair insulin signaling, directly related to mitochondrial dysfunction and insulin resistance [65, 66]. Moreover, insulin resistance can cause mitochondrial biogenesis impairment, oxidative stress, membrane depolarization, and increased dopaminergic neuronal degeneration in the SN [67]. Additionally, in db/db or HFD diabetic mice, the expression of Parkin reduced with the accumulation of the zinc finger protein Parkin interacting substrate (PARIS) and the inhibition of peroxisome proliferator-activated receptor gamma coactivator 1α (PGC1α). Thus, disruption of the Parkin-PARIS-PGC1α pathway may explain the association among mitochondrial dysfunction, insulin resistance, and dopaminergic neuronal degeneration [68]. PGC1α, a key transcriptional regulator of enzymes in mitochondrial respiration and insulin resistance, is potentially pivotal in the pathogenesis of DM and PD [69, 70] (Figure 1). Furthermore, dysfunction of PINK1-Parkin-mediated mitophagy in PD has been associated with DM [71]. Impairment of mitophagy reduces insulin secretion by β cells [72]. Therefore, there is a potential for improving mitophagy to preserve β cell function and delay DM progression [73].

### 3.4 Chronic inflammation in DM and PD

DM and PD are age-related diseases in which chronic inflammation is a factor, and a new concept “senoinflammation,” was proposed by Chung *et al.* [74]. Metabolically active tissues such as adipose tissue, muscle, the pancreas, and the liver commonly induce inflammation in aging, which may cause metabolic disorders, including obesity, insulin resistance, and DM [75, 76]. In the CNS, microglia are primary immunological cells important in regulating brain immunity and inflammation [77, 78]. In PD postmortem brains, reactive microglia are involved in the degeneration of dopaminergic neurons [79]. Under various neuroinflammatory stimuli, resting microglia can be activated and polarized into two phenotypes: classical activation of the proinflammatory M1 phenotype and alternative activation of the anti-inflammatory M2 phenotype [80]. There are elevated proinflammatory cytokines, including IL-6, TNF-α and IL-1β, in the cerebral spinal fluid (CSF), serum, and brain tissue of patients with PD. Some PD animal models have demonstrated that both M1 proinflammatory and M2 anti-inflammatory phenotypes of microglia coexist in the early stage of PD, converting to prevalence of M1 proinflammatory microglia in later stages [81]. Moreover, the blockade of neurotoxic A1 astrocytes induced by M1 microglia is neuroprotective in PD animal models [82, 83]. Intriguingly, metabolic disorders including obesity, insulin resistance, and T2DM can regulate the transition of microglia from M2 neuroprotective phenotype to M1 neurotoxic phenotype [84, 85].

**Figure 2.**
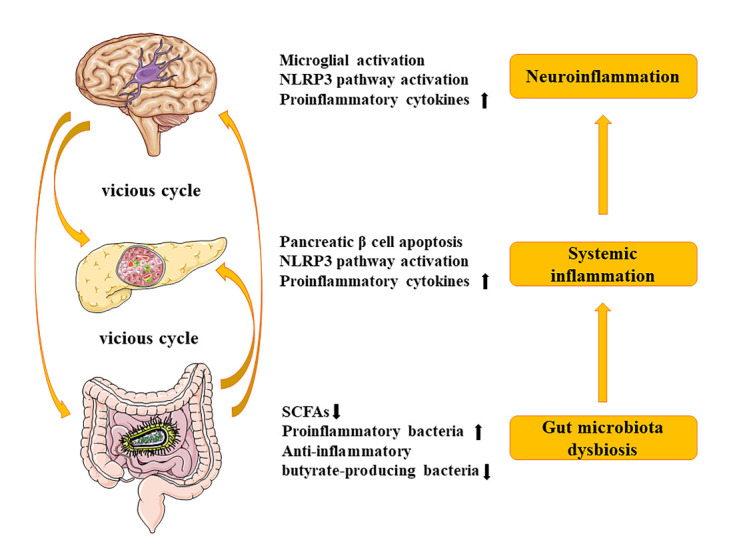
Roles of gut-brain axis in DM and PD. Gut microbiota dysbiosis causes beneficial bacterial metabolites to decrease and proinflammatory cytokine levels to increase, leading to systemic inflammation and pancreatic β-cell apoptosis. Proinflammatory cytokines then propagate from the gut-brain axis, resulting in microglial activation and NLRP3 pathway activation and further causing neuroinflammation.

The inflammasome pathway is pivotal in chronic systemic inflammation and brain inflammation, linking DM and PD [71, 86]. After sensing pathogen-associated molecular patterns (PAMPs) or danger-associated molecular patterns (DAMPs), canonical nucleotide-binding oligomerization domain- and leucine-rich-repeat- and pyrin-domain-containing 3 (NLRP3) inflammasomes assemble in the cytosol to recruit inactive pro-caspase-1 and cleave into active caspase-1, which cleaves the precursor cytokines pro-IL-1β and pro-IL-18 to generate the active cytokines IL-1β and IL-18, respectively [87]. IL-1β and IL-18 can cause systematic inflammation, impair pancreatic β-cell function, induce cell apoptosis, and exacerbate insulin resistance [88]. Then, peripheral proinflammatory stimuli activate microglial metabolic reprogramming and increase glycolysis and glutaminolysis in microglia to cause neuroinflammation, which excretes more proinflammatory cytokines initiating a vicious cycle [89, 90] (Fig. 2). Notably, mitochondrial damage plays a critical role in activating NLRP3 inflammasome. The accumulation of damaged mitochondria and an increase in mitochondrial ROS (mtROS) due to mitophagy inhibition are associated with NLRP3 inflammasome activation [91].

### 3.5 Microbiota dysbiosis of gut-brain axis in DM and PD

Currently, gut microbiota dysbiosis plays an important role in intestinal disease and in many extraintestinal diseases, including metabolic and neurodegenerative diseases [92, 93]. The gut microbiota mainly comprises six phyla, including Firmicutes, Proteobacteria, Verrucomicrobia, Fusobacteria, Actinobacteria, and Bacteroidetes [94]. Microbiota dysbiosis results in a deficiency of short-chain fatty acids (SCFAs) such as acetate, propionate, and butyrate, which are metabolites of healthy gut microbiota, and has been demonstrated in obesity, T1DM, and T2DM [95]. Some studies demonstrated that the ratio of Firmicutes to Bacteroidetes decreased in T1DM and T2DM [96, 97]. At the genus level, patients with T2DM have reduced levels of butyrate-producing bacteria, such as Bifidobacterium, Akkermansia, and Faecalibacterium, and increased levels of Dorea, which are involved in chronic inflammation [98, 99]. Similarly, the abundances of Bacteroidetes, Verrucomicrobia, and Proteobacteria were higher, whereas that of Firmicutes was lower in the feces of patients with PD. Additionally, at the genus level, putative proinflammatory bacteria, *Oscillospira* spp., *Akkermansia* spp., and *Bacteroides* spp., were significantly increased in patients with PD. In contrast, the abundance of putative anti-inflammatory butyrate-producing bacteria *Blautias* spp., *Coprococcus* spp., and *Roseburia* spp. significantly decreased in PD [100, 101]. Yan *et al.* reported that the composition of gut microbiota and metabolites in A53T monkeys showed high similarity to those in patients with PD. Therefore, metabolites mainly associated with mitochondrial dysfunction may be related to the pathogenesis of PD [102].

The mechanism linking DM and PD by microbiota dysbiosis is mainly by the gut-brain axis. In the presence of gut microbiota-caused dysbiosis, harmful metabolites and proinflammatory cytokines can damage the intestinal barrier, causing systematic inflammation and pancreatic β-cell dysfunction and further damaging the brain-blood barrier to cause neuroinflammation, immune activation, and oxidative stress [103, 104]. The gut microbiota mediates the inflammatory response based on interactions between lipopolysaccharides (LPS) produced by bacteria with toll-like receptors (TLRs) [96]. Moreover, the level of LPS in the blood was correlated with cell death, DM, and other diseases such as sepsis, inflammatory bowel disease, and neurodegenerative diseases including PD [105, 106]. Microbiota plays a significant role in the pathogenesis and progression of PD [107]. Patients with PD can exhibit microbiota-related dysbiosis and gastrointestinal complications even before the clinical onset of motor symptoms, such as hypersalivation, dysphagia, and constipation [108]. Therefore, targeting gut microbiota homeostasis with probiotic, prebiotic, synbiotic, and postbiotic (PPSP) supplementation as well as fecal microbiota transplantation (FMT) is a promising therapy for both DM and PD [109-111] (Fig. 2).

## 4. Repurposing antidiabetic drugs for the prevention and treatment of PD

**Figure 3.**
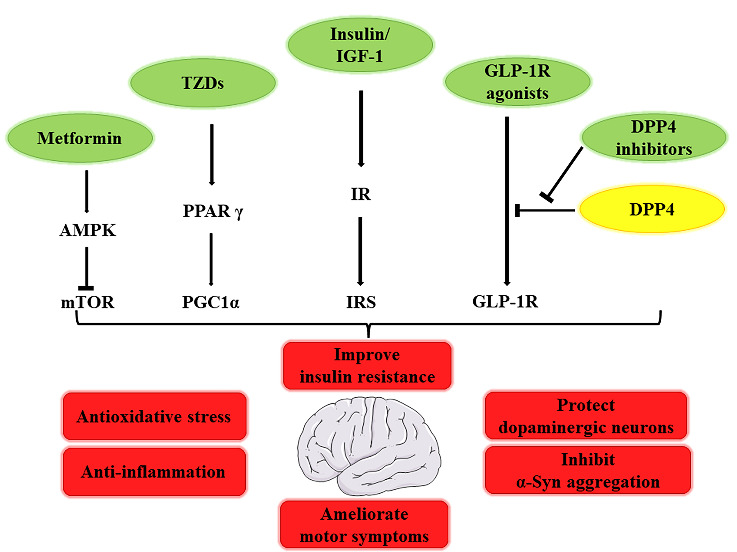
Roles of antidiabetic drugs in PD treatment. Antidiabetic drugs can be neuroprotective by improving insulin resistance. Moreover, they can inhibit α-Syn aggregation and decrease dopaminergic neuron degeneration by antioxidative stress and anti-inflammatory effects, thus ameliorating motor symptoms in animal models and patients with PD.

Currently, medication only improves the clinical symptoms of PD. There is an urgent need to discover effective drugs to delay its progression, if not cure PD. Common pathophysiological processes linking DM and PD offer a new prospective for repurposing antidiabetic drugs for the treatment of PD. Some antidiabetic drugs have antioxidative and anti-inflammatory roles, which may be neuroprotective in the prevention and treatment of PD [112] (Fig. 3).

### 4.1 Antidiabetic drugs for the prevention of PD

In patients with diabetes, some antidiabetic drugs can decrease the risk of PD. A cohort study of patients with diabetes by Brauer *et al.* showed that glucagon-like peptide-1 (GLP-1) receptor agonists or dipeptidyl peptidase 4 (DPP4) inhibitors are associated with a lower incidence rate ratio (IRR) of PD compared with other oral antidiabetic drugs (IRR 0.38; 95% CI 0.17-0.60, and IRR 0.64; 95% CI 0.43-0.88, respectively), but thiazolidinediones (TZDs) or glitazones (GTZs) do not decrease the risk of PD [113]. However, a systematic review and meta-analysis by Qin et al. suggested that GLP-1 receptor agonists decrease the risk of PD (HR 0.41; 95% CI, 0.19-0.87), while DPP4 inhibitors, GTZ, metformin, and sulfonylurea do not [114].

### 4.2 Antidiabetic drugs for the treatment of PD

Wang et al. found that the GLP-1 receptor agonist exenatide, but not pioglitazone, can ameliorate cognitive, motor, and nonmotor symptoms in patients with PD [115]. Furthermore, Jeong *et al.* suggested that DPP4 inhibitors showed beneficial effects on baseline dopamine degeneration and long-term motor symptoms in diabetic patients with PD. However, studies with large sample sizes should be conducted to prove these conclusions, which may extend the effect of antidiabetic drugs to nondiabetic patients with PD [116]. In this section, we will discuss several antidiabetic drugs used in cellular and animal models of PD as well as some current clinical trials (Table 2).

### 4.2.1 Insulin and PD

Due to the potential role of insulin resistance in PD pathogenesis, targeting insulin resistance may have therapeutic implications for PD. To study the role of insulin in the treatment of PD, researchers have focused on intranasal insulin administration to avoid hypoglycemia resulting from peripheral administration [117]. Pang *et al*. showed that intranasal insulin provided strong neuroprotection in a 6-OHDA-induced PD rat model without affecting body weight or blood glucose levels, which proved that insulin signaling may be a novel therapeutic target in PD [118]. A double-blind placebo-controlled pilot study showed that 40 international units (IUs) of intranasal insulin administered once daily for 4 weeks improved verbal fluency and motor symptoms in patients with PD or multiple system atrophy (MSA) [119]. However, a larger study with long-term observation is required to verify the role of IIS in cognitive function and motor symptoms in patients with PD [120-122].

**Table 2:** Roles of antidiabetic drugs on PD treatment.

Antidiabeticdrugs	Animal models or clinical trials	Roles in PD	Refs.
Insulin	6-OHDA-induced PD animal model	Alleviate motor deficits Protect dopaminergic neuron	[118]
	PD and MSA patients (NCT02064166)	Improve UPDRS score and H-Y scale	[119]
Metformin	MPTP-induced or rotenone-induced PD animal model	Improve motor symptoms Protect dopaminergic neuron Inhibit α-Syn phosphorylation Antioxidative stress Rescue mitochondrial function	[126-131]
	LPS-induced PD animal model	Fail to protect dopaminergic neuron	[138]
TZDs or GTZs	6-OHDA-induced or MPTP-induced or LPS-induced PD animal model	Anti-inflammation Antioxidative stress Improve mitochondrial function	[143-148]
	PD patients (NCT01280123)	Fail to improve UPDRS score	[150]
GLP-1 receptor agonists	6-OHDA-induced or MPTP-induced or LPS-induced PD animal model	Improve motor function Protect dopaminergic neuron Anti-inflammation Antioxidative stress	[154-156]
	PD patients (NCT01174810)	Improve UPDRS score Improve cognitive function	[160, 161]
	PD patients (NCT01971242)	Improve UPDRS score	[162, 163]
DPP4 inhibitors	Rotenone-induced PD animal model	Anti-inflammation Antioxidative stress	[168-172]

PD: Parkinson’s Disease; UPDRS: Unified Parkinson’s Disease Rating Scale; H-Y scale: Hoehn and Yahr scale.

### 4.2.2 Metformin and PD

Metformin is the most commonly used first-line oral antidiabetic drug belonging to the biguanide family [123]. Metformin can cross the BBB and act via AMP-activated protein kinase (AMPK)-dependent or AMPK-independent mechanisms in the CNS [124, 125]. Metformin is neuroprotective and can improve motor impairment in MPTP- or rotenone-induced PD models [126-131]. The neuroprotective role of metformin may be mediated by the inhibition of α-Syn phosphorylation and improvement of mitochondrial function [132, 133]. In SH-SY5Y and HeLa cells, as well as in wild-type C57BL/6 mice, metformin reduced the phosphorylation of Ser129 α-Syn, which was associated with the inhibition of mTOR and the activation of protein phosphatase 2A (PP2A) [134]. Moreover, metformin rescues PD phenotypes caused by hyperactive mitochondria via its role in the inhibition of the mitochondrial respiratory chain [135-137]. However, in LPS-induced PD models, metformin fails to protect dopaminergic neurons in the SN [138]. Furthermore, a meta-analysis of observational studies showed that the use of metformin in patients with DM may lead to the development of PD [139]. Thus, metformin has both beneficial and harmful effects in various PD models, particularly as a weak inhibitor of complex I of the mitochondrial respiratory chain, limiting the use of metformin for the prevention and treatment of PD.

### 4.2.3 TZDs (or GTZs) and PD

TZDs, or GTZs, are another class of oral antidiabetic drugs. TZDs are selective agonists of peroxisome proliferator-activated receptor gamma (PPARγ) that regulate glycemia and lipid homeostasis [140]. PPARγ is expressed not only in insulin-sensitive tissues but also in the SN and putamen, which expands its function in other diseases, such as PD [141]. PPARγ can activate central PGC1α signaling to exhibit promising protective effects in anti-parkinsonism therapeutics [142]. Moreover, pioglitazone and rosiglitazone have exhibited neuroprotective effects on behavioral and motor symptoms in some toxin-induced PD models, including 6-OHDA [143, 144], MPTP [145, 146], and LPS [147, 148]. Its protective roles are associated with anti-inflammation, antioxidative stress, and improvement of mitochondrial function [149]. In a phase 2, multicenter, double-blind, randomized trial of pioglitazone in early PD, 15 or 45 mg of pioglitazone per day for 1 year failed to demonstrate obvious protective effects on the UPDRS score [150]. Moreover, the translation of TZDs for PD treatment could be limited by the side effects of cardiovascular risk and its poor capacity to cross the BBB [140, 151].

### 4.2.4 GLP-1 receptor agonists and PD

Incretins, including glucagon-like peptide-1 (GLP-1) and glucose-dependent insulinotropic polypeptide (GIP), can stimulate insulin secretion by pancreatic β cells in a glucose-dependent manner, which plays an important role in insulin and glucose homeostasis [152]. GLP-1 receptor agonists, or dual GLP-1/GIP receptor agonists, have promising therapeutic potential for PD [153]. The natural GLP-1 mimetic exendin-4 has neuroprotective roles in several PD animal models [154, 155]. Moreover, Liu et al. found that other long-acting GLP-1 receptor agonists, liraglutide and lixisenatide, are superior to exendin-4 in improving motor function and protecting dopaminergic neurons in MPTP models of PD [156]. The protective mechanisms of GLP-1 receptor agonists are because of the preservation of mitochondrial function, inhibition of dopaminergic neurons apoptosis, and microglial activation [157, 158]. Several clinical trials have proven the beneficial effects of GLP-1 receptor agonists in patients with PD [159]. Aviles-Olmos et al. conducted the first clinical trial of exenatide for patients with PD. They found that exenatide can improve motor and cognitive functions, which persists even 12 months after its discontinuation [160, 161]. Moreover, Athauda et al. found that exenatide once weekly versus placebo for 48 weeks followed by a 12-week washout period can improve the UPDRS Part III score at 60 weeks. Exenatide-treated patients had augmented tyrosine phosphorylation of IRS1 and elevated expression of downstream substrates, including total Akt and phosphorylated mTOR, in serum exosomes [162, 163]. Normalizing insulin signaling and delaying disease progression make GLP-1 receptor agonists potential drugs for disease-modifying treatment [164].

### 4.2.5 DPP4 inhibitors and PD

Natural GLP-1 has a half-life of only 2-3 min and can undergo immediate degradation by DPP4. Therefore, DPP4 inhibitors can enhance the function of GLP-1 [165]. Oral DPP4 inhibitors can increase insulin secretion and decrease glucagon secretion without affecting body weight. In addition to their effect in the endocrinological system, DPP4 inhibitors have a protective role in other systems, including the cardiovascular system and CNS, due to their immunosuppressive functions [166, 167]. Abdelsalam *et al.* showed that vildagliptin reduced dopaminergic neuron degeneration and improved motor performance by targeting the receptor for advanced glycated end product (RAGE)/nuclear factor kappa B (NF-κB) anti-inflammatory pathway and Nrf-2 antioxidative pathway in rotenone-induced PD models [168]. Moreover, saxagliptin, sitagliptin, and alogliptin showed neuroprotective functions in rotenone-induced PD models [169-172]. However, gliptins cannot penetrate the BBB, whereas omarigliptin was the first gliptin able to cross it and increase GLP-1 concentration in the brain, which makes omarigliptin a potent candidate for PD treatment [173].

## 5. Conclusions

In this review, we discussed the association between PD and DM. First, epidemiological studies demonstrated that DM, or prediabetic conditions, can increase the risk of PD development, and DM can accelerate the progression of PD, including motor dysfunction and cognitive impairment. Second, we discussed the underlying pathophysiological links between PD and DM, including amyloid protein aggregation, insulin resistance, mitochondrial dysfunction, chronic inflammation, and gut microbiota dysbiosis, which may explain why patients with DM are prone to develop PD. Third, we summarized some publicly available antidiabetic drugs that show potential for repurposing in PD treatment. While the clinical safety of antidiabetic drugs has been verified, large-scale clinical trials are required to prove their effectiveness in preventing and treating PD.
